# Affect Path to Flood Protective Coping Behaviors Using SEM Based on a Survey in Shenzhen, China

**DOI:** 10.3390/ijerph17030940

**Published:** 2020-02-03

**Authors:** Jing Huang, Weiwei Cao, Huimin Wang, Zhiqiang Wang

**Affiliations:** 1 State Key Laboratory of Hydrology-Water Resources and Hydraulic Engineering, Hohai University, Nanjing 210098, China; j_huang@hhu.edu.cn; 2 Institute of Management Science, Business School, Hohai University, Nanjing 211100, China; cww@hhu.edu.cn (W.C.); wzqzjc@163.com (Z.W.)

**Keywords:** protective coping behaviors, flood risk perception, socio-demographic factors, affect path, structural equation model

## Abstract

The initial concept of flood control has gradually shifted to flood risk management which emphasizes more public participation. Therefore, understanding the public’s protective coping behavioral patterns to floods is significant, and can help improve the effectiveness of public participation and implementation of flood-mitigation measures. However, the quantitative effect of socio-demographic factors on flood risk perception and behaviors is not clear. In this study, the socio-demographic factors are included to explore the quantitative relationship with and the affect path to flood protective coping behaviors with socio-demographic factors are studied. Shenzhen City in China is chosen as the study area, which suffers frequent urban floods every year. Questionnaire surveys are conducted in five flood-prone communities there, and 339 valid questionnaires were collected. The correlations between flood risk perception, flood risk knowledge, flood risk attitude, socio-demographic factors, and protective coping behaviors are analyzed firstly. A structural equation model (SEM) about these factors is then established to verify the correctness of hypothetical paths and discover new paths. The results indicates that socio-demographic factors and flood risk perception do not have impacts on protective coping behaviors directly, but are mediated by flood risk knowledge and flood risk attitude. Flood risk attitude is an important factor that affects protective coping behaviors directly. Moreover, two affect paths to flood protective coping behaviors are proposed. The findings of Shenzhen city in this study can be extended to other cities with similar characteristics, providing support for conducting effective flood mitigation measures.

## 1. Introduction

With the change of global climate, the frequency and intensity of urban floods have increased significantly [1], which will affect the normal operation of cities seriously and threaten the public safety and economic development of cities simultaneously [2]. In recent years, the scale of evacuation due to floods has shown a rapid increase worldwide. Floods triggered by heavy rain in Louisiana, U.S., caused the emergency evacuation of approximately 18,000 people and destroyed thousands of homes in 2016. In China, the problem of urban flooding is more prominent. In 2018, northern Anhui Province, China suffered severe floods, and more than 100,000 people were relocated, and a total of 2.632 million people were affected. In order to reduce the impact of floods on the public, the shift to risk-based flood management approaches is happening worldwide, so a deep understanding of flood mitigation behavior is considered important [3]. Group-based coping behaviors have proved to be capable of significantly reducing flood-related damage [4]. Therefore, how to guide public in the flood prone areas to adopt correct and appropriate protective coping behaviors will become a key part of the risk-management process.

The study on disaster coping behaviors in floods began in 1970s, by Huerta and Horton [5], which aimed to test hypotheses that “older persons report greater material and personal loss from floods than younger persons”. Subsequently, what factors could encourage to develop protective coping behaviors had become a hotspot of research. Initially, most researchers focused on the impact of public self-attribution on coping behaviors, such as age, economic status, gender, education etc. Regarding the different impact of self-attributes on behaviors, elderly people, or people who had lived in a place for a long time, were more aware of potential flood risks and were more likely to take protective actions [5,6,7]. People with lower income levels were more likely to suffer greater flood losses, reflecting that economic status determined the initiative of flood coping behaviors [8]. With regard to education, higher education levels were strongly associated with positive coping behaviors [7,8]. As for gender, a study in Serbia [9] found that females tended to adopt effective protective behaviors during disasters. Although the public’s self-attribution was very easy to obtain, many studies only considered the correlation between factors, without in depth research. Then public’s mastery of emergency knowledge was considered to be closely related to the occurrence of response behaviors. Knowledge was the basis for people to act in response to disasters. Often, flood experiences were considered direct factors that affected protective coping behaviors. Nevertheless, flood experiences essentially were transformed into flood knowledge of exposure, which actually affected behavior [10]. With the continuous improvement of psychological theory, more attention had been paid to psychological factors, such as risk perception emotions and attitudes. For risk perception, the relationship between flood risk perception and protective behaviors was widely recognized because of the application of mature theory, such as protection motivation theory (PMT) [11,12]. A higher level of risk perception was believed to help the public to take correct and timely mitigation actions [13]. The public’s emotions and attitudes would strongly influence the action taken to deal with the disaster, because the adoption of behaviors was often based on objective emotional perception [14]. In terms of worrying, worried emotions would promote the public taking preventive measures, according to published studies [15,16]. Besides, trust in flood protection [16,17] was considered a key factor that affected protective coping behaviors.

Based on those studies of influencing factors of flood coping behaviors, what has been more studied is a focus on the relationship between these factors. PMT was widely used in disaster risk management and emergency preparedness [18], which was able to explain and predict factors that could not be directly measured, such as public risk perception. Under PMT, some interesting conclusions had indeed been found, such as the positive and direct relation between risk attitudes and protective coping behaviors [17]. However, inconsistent results had also emerged; for example, Liu and Jiao [19] found a positive direct impact between risk perception and protective coping behavior, while Lemée et al. [20] found a moderating variable (anxiety-state, place identity) between the two, indicating an indirect relation. In addition, PMT did not account for the features of people themselves (age, education, income, et al.), which had been “empirically documented and theoretically framed within socio-environmental psychology for over 20 years” [20] and were important for analyzing differences in people with different characteristics. A social-cognitive model was a good solution to the deficiency of PMT, because it included social background factors, risk perception and protective coping behaviors, and was also capable of analyzing the interaction between factors. Lee and Lemyre [21] proposed a social-cognitive model based on a large number of studies on terrorism, and then was widely applied in disaster risk management.

Although many studies have been focused on the mechanism of protective coping behaviors against floods, the quantitative effect of socio-demographic factors on flood risk perception and behaviors and the affect path to flood protective coping behaviors with socio-demographic factors are not clear. Therefore, socio-demographic factors are introduced in order to detect the new mechanism of protective coping behaviors. Shenzhen City in China, which suffers frequent floods every year, is selected as our study area. A structured questionnaire is conducted in five flood-prone communities there. Based on the questionnaire surveys, the correlations between flood risk perception, flood risk knowledge, flood risk attitudes, socio-demographic factors, and protective coping behaviors are analyzed. Then a structural equation model is established to verify the correctness of hypothetical paths and discover new paths of flood risk perception, flood risk knowledge, flood risk attitude, socio-demographic factors, and protective coping behaviors. 

## 2. Materials and Methods

### 2.1. Study Area

Shenzhen, located in the south of Guangdong Province, is one of four alpha cities (GaWC 2018) and the first Economic Special Zone in China. It covers an area of 1996.85 km^2^ and 9 districts is under its jurisdiction, including a new district. By 2018, the total resident population was 13.025 million and the regional GDP had reached 2422.198 billion yuan in 2018. Shenzhen lies on the east bank of the Zhujiang River estuary, bordering Daya Bay and Dapeng Bay, and therefore it is a typical coastal developed city. The climate in the city belongs to subtropical maritime climate, with the annual precipitation of 1933.3 mm. Due to the location of the coastal area and the humid climate, Shenzhen is a frequent area of typhoon activity, and vulnerable to heavy rains and severe floods [22]. As a consequence, when typhoons or rainstorms occur, urban waterlogging will form in a quite a short time. Coupled with the accumulation of social wealth in the urban areas, the economic losses caused by flood disasters are often very high. According to the statistics, Shenzhen’s economic losses caused by floods in 2014 exceeded 254 million yuan. Even more frightening, some experts [23] predict that this figure will surpass 257 million yuan by 2020 and 3.09 million in 2028. For the foreseeable future, Shenzhen will still be a Chinese “Big City” under the threat of flood disasters, with huge potential economic losses.

**Figure 1:**
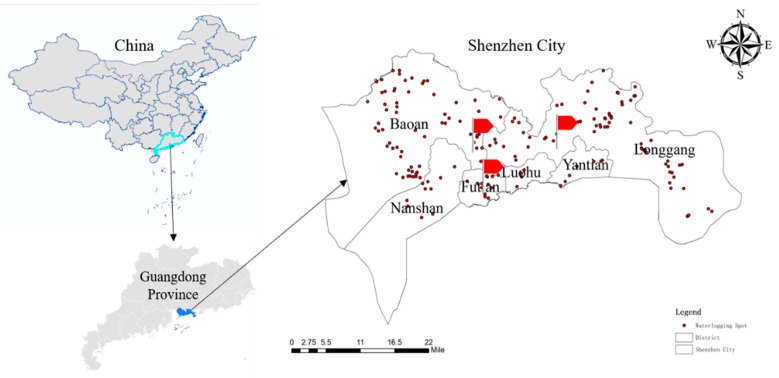
Study area.

In order to ensure a better investigation result, this paper selected the specific research community based on the waterlogging spot data of Shenzhen. Simultaneously, considering that there was no flooding in high-rise residential buildings, we finally chose five low-rise communities near the easily flooded areas in three different districts (Figure 1).

### 2.2. Methods

The path of flood risk perception, socio-demographic factors, flood risk knowledge, flood risk attitude, protective coping behaviors is discovered and quantified by following these steps below. 

(1) Hypotheses

PMT revealed the process of accepting information, perceiving risk, and finally leading to protective coping behaviors. This work considers flood risk perception, flood risk knowledge, and flood risk attitude as influencing factors according to the perceived risk process in PMT, which is divided into two parts: threat appraisal and coping appraisal. Threat appraisal often relies on individual knowledge of flood risk and risk perception of actual conditions. Flood risk attitude reflect people’s assessment of their ability to overcome threats, that is, coping appraisal in PMT. However, PMT do not consider the impact of socio-demographic factors on protective coping behaviors, while the social-cognitive model do. Therefore, socio-demographic factors are added to the hypothesis in this study.

Hypotheses about paths also follow the framework of PMT and social-cognitive model. When the public perceives the risk of flooding, they unknowingly acquire some flood risk knowledge, such as what self-rescue measures are available. Therefore, we assume that higher risk perception will be associated with higher levels of flood risk knowledge (H1). The public’s perception of flood risk represents their self-evaluation of the potential risks they face. The perception has an impact on public risk-taking attitude, which results in a shift in attitude [24]. In general, a higher level of risk perception will lead to a more positive risk attitude (H2). For flood risk perception, socio-demographic factors are considered as differentiating factors that produce the performance of risk perception differences among different people. As the work of Bustillos Ardaya, Evers, and Ribbe [25], flood risk perception is influenced by demographic factors principally and directly. So, socio-demographic factors are predicted to be related to flood-risk perception (H3). This is because the grasp of flood risk knowledge had, to a certain extent, determined people’s confidence in self-efficacy, according to the PMT theory. So, flood risk knowledge is possibly a potential factor influencing individuals’ attitudes towards floods (H4). According to Xu et al. [26], disaster risk perception is found to be significantly positively correlated with coping behaviors to avoid disasters. At the same time, considering the important role of flood risk perception in flood risk management, we expect a higher risk perception to be directly related to more positive coping behaviors (H5). Good pre-disaster education and mitigation training is an important preventive measure that prompts people in the flood-prone areas to take effective responses to flood risk. In fact, it confirms the positive effect of flood risk knowledge on protective coping behaviors. Besides, according to the work by Zhang et al. [27], people with new knowledge and updated information are more likely to respond effectively to typhoons. Therefore, we expect that flood risk knowledge have a positive effect on protective coping behaviors (H6). The public’s attitudes to flood risk reflect a willingness to respond to floods. Lee et al. [28] also find out that coping behaviors are significantly influenced by the attitudes to climate change. Therefore, in this study, protective coping behaviors are assumed to be influenced by flood risk attitude positively (H7). With regard to protective coping behaviors, socio-demographic factors are also the factors that cannot be ignored, and recent studies [29] have been devoted to finding out how some basic attributes of people had an impact on the disaster reduction behaviors. Therefore, socio-demographic factors are also expected to be positively related to flood risk perception and protective coping behaviors (H8).

(2) Descriptive Statistics and Correlation Analysis

After collating the questionnaire data, descriptive statistics are applied to quantitatively describe and summarize the features of the socio-demographic characteristics of the respondents. Then, the correlation is measured between protective coping behaviors and socio-demographic factors, flood risk perception, flood risk knowledge and flood risk attitude, using IBM SPSS 22.0 (SPSS Inc., Chicago, IL, USA). In this step, the degree of association between socio-demographics, flood risk knowledge, flood risk attitude, protective coping behaviors are examined by Equations (1) and (2). Then, the correlation between each variable are preliminarily summarized in order to observe the difference in correlation.

*Cov* = E[*XY*]−E[*X*]E[*Y*] (1)

(2) $$r\left(X,Y\right)=\frac{Cov\left(X,Y\right)}{\sqrt{Var\left[X\right]Var\left[Y\right]}}$$

In Equation (1), E[*X*] and E[*Y*] is the expected values of *X* and *Y*. In Equation (2), *Cov*(*X*, *Y*) is the covariance of *X* and *Y*, *Var*[*X*] is the variance of *X*, and *Var*[*Y*] is the variance of *Y*.

(3) Path Analysis

**Figure 2:**
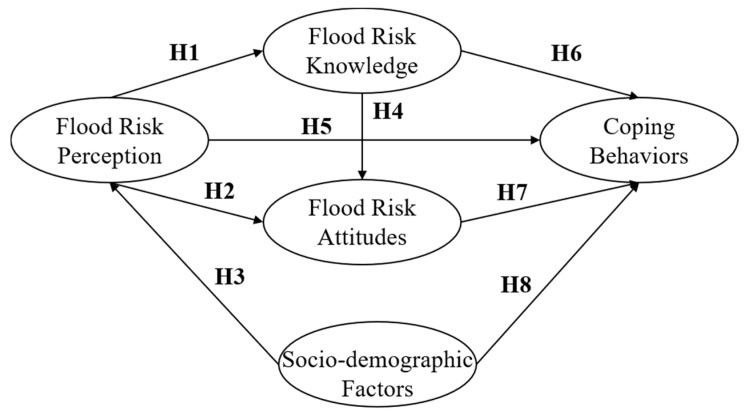
Path hypothesis of flood risk perception, flood risk knowledge, flood risk attitudes, protective coping behaviors, and socio-demographic factors.

Based on path hypothesis in Figure 2, a structural equation model (SEM) about flood risk perception, socio-demographic factors, flood risk knowledge, flood risk attitude, and protective coping behaviors is established. Then, the paths which reflect how socio-demographic factors, flood risk perception, flood risk knowledge, flood risk attitude affect behaviors are verified through IBM AMOS 24.0 (SPSS Inc., Chicago, IL, USA). In this process, the influence mechanism among various variables are found through Equations (3)–(5) by path analysis, with special attention to the relationship between socio-demographic factors and flood risk perception on protective coping behaviors.

*Y* = Λ*_y_η* + *ε* (3)

*X* = Λ*_x_ξ* + *σ* (4)

Equations (3) and (4) are measured models, *X* is the observed variable of the exogenous latent variable, *Y* is the observed variable of the endogenous latent variable, Λ*_x_* is the correlation coefficient matrix of the exogenous latent variable and its observed variable, Λ*_y_* is The matrix of the correlation coefficient between the latent variable and its observed variable, *σ* is the residual of the exogenous equation, and *ε* is the residual of the endogenous equation.

*η* = B*η* + Γ*ξ* + *ζ* (5)

Equation (5) is a structural model, *η* is an endogenous latent variable, *ξ* is an exogenous latent variable, B is a correlation coefficient between endogenous latent variables, Γ is a correlation coefficient between an exogenous latent variable and an endogenous latent variable.

### 2.3. Questionnaire Design

The purpose of the questionnaire survey is to understand the relationship between flood risk perception and protective coping behaviors.

The overall content of the questionnaire follow the framework of the PMT and social-cognitive model which mainly include social elements, emotion, cognitive factors and behaviors [21,30]. Under this framework, the content of the survey is mainly divided into five sections (Table 1) which are socio-demographic factors, flood risk perception, flood risk knowledge, flood risk attitude and protective coping behaviors [31]. Each section contains several specific, measurable factors. The first section includes 4 important factors that affect the public protective coping behaviors, such as gender [9], age [32], education [33], income [34] and location. The second section which contains flood experience [32,33], knowledge of flood types et al., aims to assess the flood risk knowledge of respondents. The third section is measured by trust [16] and worry [15,16]. The fourth section focuses on how to evaluate the validity of public protective coping behaviors and consisted of preparation of supplies before a disaster, willingness to collect flood information, understanding of disaster-prevention measures, insurance willingness. The last section aims to test the public’s self-assessment of their level of flood risk perception.

Ultimately, the questionnaire of this study is designed into a structured questionnaire and adopted a 5-point Likert scale to facilitate the collation and standardization of the data. The Likert scale is commonly used to investigate respondents’ attitudes or opinions on certain things or events [35], which is suitable for this research object. In order to ensure the understanding of the questions in the questionnaire, before the formal investigation some respondents are chosen to verify that the problem is easy to understand. Simultaneously, the reliability and validity of the questionnaire are preliminarily tested on that small sample.

## 3. Results

### 3.1. Statistical Result

In this study, 339 valid survey questionnaires from 5 communities in three different districts of Shenzhen are analyzed in detail. The Cronbach’s alpha of questionnaires reaches 0.811, which representes a high level of reliability [36]. Table 2 summarizes the socio-demographic attributes of gender, age, education and income in the study area.

Of the 339 respondents, 89 (26.25%) are from Xixiang District, 72 (21.24%) are from Shatou District, and 178 (52.51%) are from Nanwan District. Among the respondents, the percentage of male and female are very close, accounting for 50.74% and 49.26%, respectively. The majority of respondents are aged from 21 to 40 years old accounting for 44.84% and 28.02%, respectively. With regard to the education level, no respondents receives master’s degree or higher, and most of the respondents are high school educated (36.28%). In terms of income, the proportion of respondents earning less than 10,000 yuan every month reaches 88.79% while only 1.48% earn more than 30,000 yuan. In general, the characteristics of respondents in three districts are almost identical.

### 3.2. Correlation Results

A correlation matrix is presented in Table 3 for the all variables. The results show that there are correlations between flood risk perception, socio-demographic factors, risk knowledge risk attitudes and protective coping behaviors, but the degrees of correlation vary.

(1) About Socio-Demographic Factors

With regard to the correlation between socio-demographic factors and flood risk perception, out of 4 correlation coefficients (4 indicators of socio-demographic factors and 1 indicator of flood risk perception form a 4 × 1 correlation matrix), 2 are significant at the 0.01 level (2 of 4 coefficients were significant, 50.00%). Education (*r* = 0.23) and location (*r* = 0.15) prove to be positively related to flood risk perception. 

In terms of socio-demographic factors and risk knowledge, out of 20 correlation coefficients (4 indicators of socio-demographic factors and 5 indicators of risk knowledge form a 4 × 5 correlation matrix) between socio-demographic factors and flood risk knowledge, 6 are significant at the 0.01 level and 2 are significant at the 0.05 level (total 40.00%). Flood experience utility is related to age (*r* = −0.11) and education (*r* = 0.16) of respondents. Knowledge of flood types, knowledge of flood is found to be relevant for education (*r* = 0.16, *r* = 0.25) and location (*r* = 0.24, *r* = 0.15). Knowledge of flood damage is related to education (*r* = 0.12) while knowledge of self-help measures is related to location (*r* = 0.15). 

With regard to protective coping behaviors, socio-demographic factors and protective coping behaviors form a 4 × 4 correlation matrix with a total of 16 correlation coefficients. Of these correlation coefficients, 5 (31.25%) have proven to be relevant. Understanding of disaster prevention measures is correlated to age (*r* = 0.11) and education (*r* = 0.12). Insurance willingness of public is related strongly to age (*r* = −0.19), education (*r* = 0.33) and income (*r* = 0.14). Although not all the indicators are related to protective coping behaviors, it is undeniable that a potential relationship should to be studied between socio-demographic factors and protective coping behaviors.

Therefore, socio-demographic factors are related with flood risk perception, flood risk knowledge and protective coping behaviors. However, the degree of correlation between these factors vary, and the correlation with risk perception is considered the highest compared with other factors.

(2) About Flood Risk Perception

A total of 85.71% (6 of 7 coefficients are significant) of correlation coefficients of flood risk perception and flood risk knowledge, flood risk attitudes are significant at the 0.01 level (Table 3). Moreover, the most notable of these is the significant positive correlation of knowledge of flood causes with self-assessment of flood risk perception (*r* = 0.52). The results show that there is a close relation between flood risk perception and flood risk knowledge, flood risk attitudes.

In term of flood risk perception and protective coping behaviors, the impacts of flood risk perception on preparation of supplies before disaster (*r* = 0.25), willingness to collect flood information (*r* = 0.34), understanding of disaster prevention measures (*r* = 0.43), and insurance willingness (*r* = 0.20) are proved to be reasonable through correlation analysis. Because all the correlation coefficients (1 indicator of flood risk perception and 4 indicators of protective coping behaviors form a 1 × 4 correlation matrix, 4 of 4 coefficients are significant, 100.00%) are significant at the 0.01 level, flood risk perception is found to be related with protective coping behaviors.

Therefore, in addition to socio-demographic factors, flood risk perception is also closely related to flood risk knowledge, flood risk attitudes, and protective coping behaviors.

(3) About Protective Coping Behaviors

There is a strong link between flood risk knowledge and protective coping behaviors (5 indicators of flood risk knowledge and 4 indicators of protective coping behaviors form a 5 × 4 correlation matrix, and 19 of 20 coefficients are significant, 95%). In the correlation matrix, the number of correlation coefficients exceeding 0.3 reaches 10 and all these coefficients are significant at the 0.01 level. Only the correlation between knowledge of self-help measures and insurance willingness is not significant.

The correlation of flood risk attitude and protective coping behaviors is also obvious (2 indicators of flood risk attitude and 4 indicators of protective coping behaviors form a 2 × 4 correlation matrix, 7 of 8 coefficients are significant, 87.5%). Preparation of supplies before disaster is in connection with both worry (*r* = 0.15) and trust (*r* = 0.30). Willingness to collect flood information is influenced by the level of both worry (*r* = 0.11) and trust (*r* = 0.34). The correlation between insurance willingness and worry (*r* = 0.16), trust (*r* = 0.20) is also significant. In terms of understanding of disaster prevention measures, purely related to trust are (*r* = 0.13). On the whole, all the indicators of protective coping behaviors except understanding of disaster-prevention measures are linked strongly with flood risk attitude.

From the results above, we draw the following conclusion: flood risk knowledge, flood risk attitude are connected closely with protective coping behaviors. Considering that socio-demographic factors and flood risk perception are also related to protective coping behaviors, we find out that all the factors in this study have an impact on the occurrence of the public protective coping behaviors when facing flooding.

(4) Summary

**Figure 3:**
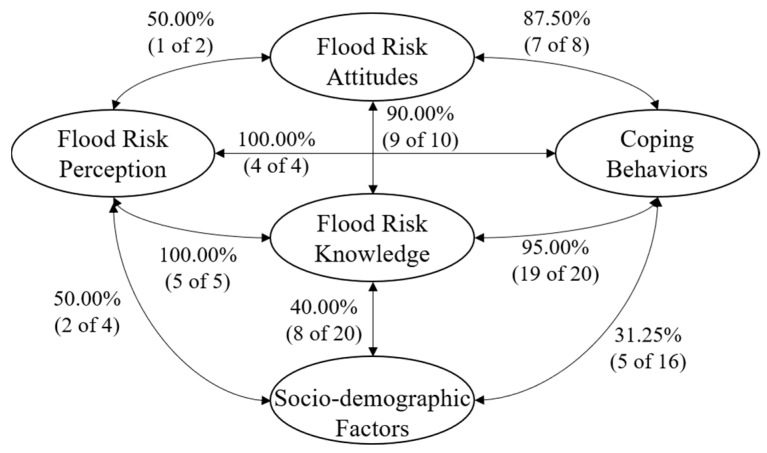
Summary of the correlation between factors.

As hypothesized, there is a complex correlation between flood risk perception, socio-demographic factors, flood risk knowledge, flood risk attitude, and protective coping behaviors (Figure 3). The correlation between flood risk perception, flood risk knowledge and protective coping behaviors is highest. Socio-demographic factors have the lowest correlation with protective coping behaviors. Although correlation analysis is able to find the connection between factors, it cannot discover the influence path. In other words, the correlation between the two factors is possibly due to the existence of indirect effects, rather than the direct influence of the variables. For example, Grahn and Jaldell [37] have proved the link between flood risk perception and flood risk knowledge, and we discover that flood risk knowledge is closely related to protective coping behaviors. So, it believes that the relationship between flood risk perception and protective coping behaviors is not that straightforward. How exactly flood risk perception, socio-demographic factors, flood risk knowledge, flood risk attitudes and protective coping behaviors interact is to be further proved by path analysis in the next section.

### 3.3. Path Model Results

#### 3.3.1. Model Fitting Result

Different fit indices are examined to evaluate the fit of the path model [20]: Chi square to df ratio (χ^2^/*df*), the root mean square error of approximation (RMSEA), the goodness-of-fit index (GFI), the comparative fit index (CFI) and the Tucker–Lewis index (TLI). The results (Table 4) of model fit suggest that the final model which supported socio-demographic factors and flood risk perception having an indirect impact on protective coping behaviors, is consistent with the reality.

#### 3.3.2. Analysis of the Impact Degree

**Figure 4:**
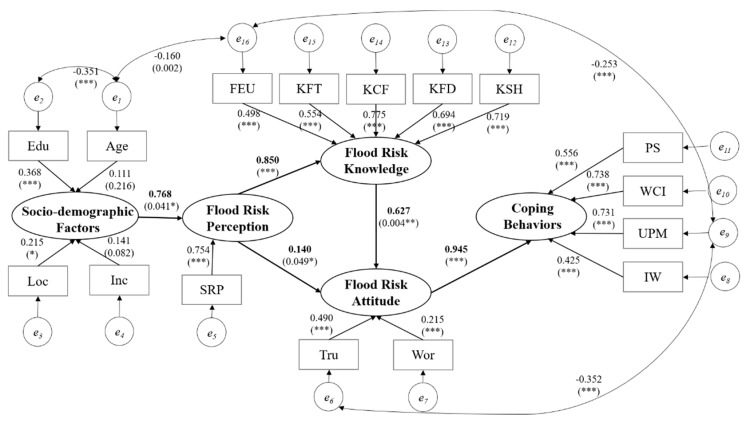
Path modeling results. Note: * *p* < 0.05, ** *p* < 0.01, *** *p* < 0.001.

Different paths, according to correlation in Figure 3, are tested for rationality. The final path model which contains 6 paths in total is shown in Figure 4. Compared with the initial hypothesis, H5, H6, H8 are rejected through the following results.

(1) Direct Effects

Direct effects describe the direct relationships between variables. As Figure 4 depicts, the direct impact of flood risk attitude on protective coping behaviors is considered to be the largest, reaching 0.945 (*p* < 0.001), indicating that flood risk attitude the most closely related factor to protective coping behaviors. Flood risk perception is closely related to flood risk knowledge (0.850, *p* < 0.001), which revealing that the improvement of flood risk perception will significantly promote the process of learning and acquisition of flood risk knowledge. The influence of socio-demographic factors on flood risk perception is ranked third (0.768, *p* < 0.05), which have the similar effect as flood risk knowledge on flood risk attitude (0.627, *p* < 0.01). Although the relation between flood risk perception and flood risk attitude is not significant in this structural equation model (0.140, *p* < 0.05), this path is still preserved on account of the relative possibilities between these two factors and is supported by many researches, such as Fahad (2018) et al. [38].

(2) Indirect Effects

One advantage of the structural equation model is that it can measure the indirect effects between different variables. As shown in Table 5, socio-demographic factors are certificated to have indirect effects on flood risk knowledge (0.653), flood risk attitudes (0.517), and protective coping behaviors (0.489). Simultaneously, flood risk attitudes (0.533) and protective coping behaviors (0.636) are indirectly affected by flood risk perception. Besides, an indirect link between flood risk knowledge and protective coping behaviors (0.593) is also found in this path model. The indirect effects between variables prove that the relations between socio-demographic factors, flood risk perception and protective coping behaviors are not direct.

(3) Total Effects

Total effect reflects the degree of interaction between various factors. As can be seen from Table 6, socio-demographic factors, flood risk perception, flood risk knowledge, and flood risk attitude all have impacts on protective coping behaviors, but different factors have different effect degrees. Flood risk attitude have the greatest total effect (0.945) on protective coping behaviors, and the total effect is consistent with the direct effect because there is no mediator variable in the path. The total effects of flood risk perception (0.636), flood risk knowledge (0.593) on protective coping behaviors are both more than 0.5, which indicate that these two factors have a significant impact on protective coping behaviors. Socio-demographic factors (0.489) exert minimum influence on protective coping behaviors, comparing to other variables, but the relationship between them is complex as consequence of multiple mediator variables.

#### 3.3.3. Affect Path to Protective Coping Behaviors

Firstly, the results indicate that socio-demographic factors have an indirect and positive effect on protective coping behaviors, rather than a direct impact. There are 3 mediator variables (flood risk perception, flood risk knowledge, and flood risk attitude) which form 2 different paths between socio-demographic factors and protective coping behaviors:

1. Socio-demographic factors → flood risk perception → flood risk knowledge → flood risk attitudes → protective coping behaviors.
2. Socio-demographic factors → flood risk perception → flood risk attitude → protective coping behaviors.

For path 1, socio-demographic factors affect the public flood risk perception firstly, then influence the acquisition of flood risk knowledge which leads to flood risk attitude changing, and in the end, produce the change of protective coping behaviors. Although, the direct link of flood risk knowledge and protective coping behaviors is not found in this path model, the path between flood risk knowledge and flood risk attitude still explain why flood risk knowledge is related strongly to protective coping behaviors. In path 2, socio-demographic factors also affect flood risk perception first, but compared with path 1, it directly influences flood risk attitudes, and ultimately make individual protective coping behaviors different.

**Figure 5:**
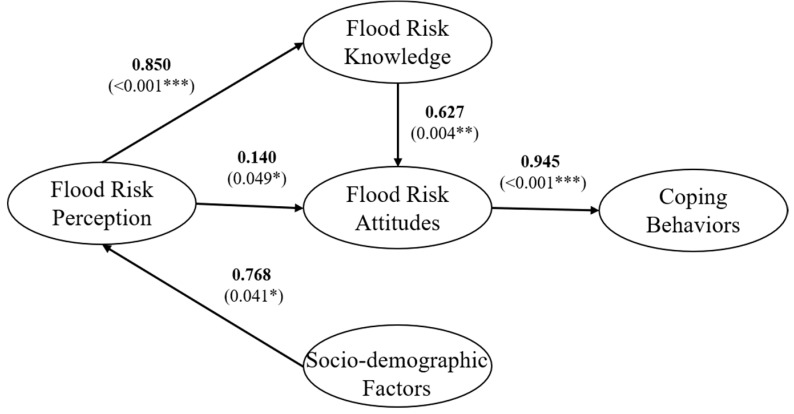
Affect path to protective coping behaviors. Note: * *p* < 0.05, ** *p* < 0.01, *** *p* < 0.001.

Secondly, we find out that flood risk perception have an indirect impact on protective coping behaviors as expected. The path model do not confirm the direct relation between flood risk perception and protective coping behaviors, but two mediating variables (flood risk knowledge and flood risk attitudes) are found in this relationship. Through the comparison of the above path 1 and path 2, it is found that the indirect effects of socio-demographic factors on protective coping behaviors rely on the indirect influence of flood risk perception on protective coping behaviors. Because both paths are based on the impact on flood risk perception, as Figure 5 shows, which explain why different socio-demographic characteristics of the public display different levels of flood risk perception and protective coping behaviors.

## 4. Discussion

In this paper socio-demographic factors are added into the SEM as a latent variable for the first time, integrating PMT and social-cognitive models. This study extends the effect of socio-demographic factors on flood protective coping behaviors and then proposes a new model to make the internal action mechanism of protective coping behaviors.

In this paper, we find that socio-demographic factors, such as age, education, income, location do not have direct impact on public protective coping behaviors. At the beginning of the study of coping behavior, socio-demographic factors are considered as important factors. Through statistical analysis, many studies [32,33,34] have found that socio-demographic factors play a non-negligible role on people’s behaviors in the face of disasters. However, these studies tend to assume a direct link, which means that the impact of age, gender, income, location and education level on the public’s behavior is direct. In fact, the relationship between coping behavior and socio-demographic factors is not so simple. As the study in this paper finds, socio-demographic factors do not have a direct impact on coping behavior, but there are two different, indirect paths of action. These two paths between socio-demographic factors and protective coping behaviors, contain in total three mediation variables including flood risk perception, flood risk knowledge and flood risk attitude with the total effect of 0.489 (Table 6). Besides, what socio-demographic factors really directly affect is public perception of risk, and this finding is in line with the studies conducted by Wang et al. [14] and Kellens et al. [39]. In psychological experiments, different personalities are found to make different in degrees of risk perception [40], and individual personality is influenced by age, education and so on, which explains why socio-demographic factors are closely related with flood risk perception instead of protective coping behaviors.

We also find that the influence of flood risk perception on protective coping behaviors is indirect, flood risk knowledge is found to be mediating variable between them. However, similar work in French coastal cities [20] also finds an indirect link between flood risk perception and protective coping behaviors, but they ignore the impact of flood knowledge on the choice of coping strategy. That is because the people there can acquire sufficient flood risk knowledge and coping skills through training and education provided by the government. However, in Shenzhen, the community’s flood preparedness and response plan are incomplete, and the public flood risk knowledge is deficient. Also, because of the extremely low proportion of flood prevention and mitigation training, the public’s protective coping behavior relies on past experience and the willingness to take action, that is flood knowledge. Therefore, flood risk perception affects flood risk knowledge firstly, and then flood risk attitude, and lastly protective coping behaviors. Therefore, flood risk knowledge plays important roles in the mechanism of flood protective coping behaviors in these cities lacking of effective training and education program. Although different characteristics of study areas will lead to different paths between flood risk perception and coping behavior, the work in Shenzhen is of practical significance. Because many cities still have the same characteristics and problems as Shenzhen. The findings of this work can be extended, providing support for conducting effective training and education.

Besides, flood risk attitude is found to be the only direct factor that affects individual protective coping behavior, with the maximum impact (0.945). There is no doubt that risk attitudes have a significant and direct impact on the protective coping behavior of the public, which is consistent with most published studies. For example, Viglione et al. [41] formed a conclusion that risk-taking attitude accounted for the public performance away from dangerous sources, by the means of socio-hydrology modelling. Worry and trust, as observable parts of risk attitudes, had also proven to be the direct predictors of protective coping behavior according to the study in Europe [42]. Therefore, we believe that guiding the public to form a positive attitude towards flood risks will help the public to take positive and effective actions to deal with floods, and minimize the damage during the disaster.

Although the fitting results of the model are encouraging, this study still has some limitations. Firstly, some respondents were still too cautious and avoided choosing extreme answers, which might cause some uncertainty in the final results. Secondly, there might be other factors that we ignore, which affected the protective coping behaviors of individual. Thirdly, it is regrettable that there is still a correlation between the observed variables, such as age and gender, which may cause the final results to deviate from the ideal situation.

## 5. Conclusions

This study investigates different typical communities in Shenzhen City and obtained data through questionnaires. After analyzing the correlation among various factors, a structural equation model is established to detect the effect of socio-demographic factors on flood protective coping behaviors and further study the internal mechanism of protective coping behaviors. In conclusion, findings in this study are:

1. This study adds socio-demographic factors as latent variables to the SEM about protective coping behaviors. We find out that socio-demographic factors had an indirect and positive effect on protective coping behaviors including three mediating variables (flood risk perception, flood risk knowledge and flood risk attitude).
2. Two paths between socio-demographic factors and protective coping behaviors are found:
  (1) Socio-demographic factors → flood risk perception → flood risk knowledge →flood risk attitude → protective coping behaviors.
  (2) Socio-demographic factors → flood risk perception → flood risk attitude → protective coping behaviors.
3. Different factors have different influence degrees on protective coping behaviors. The most influential factor is flood risk attitude (total effects reach 0.945), followed by flood risk perception (total effect of 0.636). The third one is flood risk knowledge, with the total effect of 0.593. Compared with other factors, socio-demographic factors have less influence on protective coping behavior, with the total effect of 0.489.
4. Although different affect paths between socio-demographic factors and protective response behaviors can be found in different study areas, the findings of Shenzhen City are of general significance. This is because there are many cities that have the same characteristics as Shenzhen, they lack a perfect community flood preparedness and response plan, and the public flood risk awareness is still low. The affect paths can be extended to those cities, with which the trend of public protective coping behaviors can be assessed using accessible demographic data, and the effectiveness of flood prevention and mitigation training can be improved.

## Figures and Tables

**Table 1:** The contents of questionnaire.

Section	Variables	Code	Final Value Used in Analysis
Socio-demographic factors	Gender	Gen	Male = 0, female = 1
	Age	Age	Number of years
	Education	Edu	Middle school and below = 1, High school = 2, Bachelor = 3, Master = 4, Doctor = 5
	Income per month	Inc	<¥5000 = 1, ¥5000–10,000 = 2, ¥10,000–20,000 = 3, ¥20,000–30,000 = 4, >¥30,000 = 5
	Perception of location flooding possibility	Loc	5-point scale: unlikely = 1, most likely = 5
Flood risk knowledge	Flood experience utility	FEU	5-point scale: useless = 1, most useful = 5
	Knowledge of flood types	KFT	5-point scale: unknown = 1, know well = 5
	Knowledge of flood causes	KCF	5-point scale: unknown = 1, know well = 5
	Knowledge of flood damage	KFD	5-point scale: unkown = 1, know well = 5
	Knowledge of self-help measures	KSH	5-point scale: unknown = 1, know well = 5
Flood risk attitudes	Worry	Wor	5-point scale: not worry = 1, very worry = 5
	Trust	Tru	5-point scale: not trust = 1, very trust = 5
Protective coping behaviors	Preparation of supplies before disaster	PS	5-point scale: not prepare = 1, prepare well = 5
	Willingness to collect flood information	WCI	5-point scale: unwilling = 1, very willing = 5
	Understanding of disaster prevention measures	UPM	5-point scale: unkown = 1, know well = 5
	Insurance willingness	IW	5-point scale: unwilling = 1, very willing = 5
Flood risk perception	Self-assessment of flood risk perception	SRP	5-point scale: worst = 1, best = 5

**Table 2:** The Socio-demographic variables of the respondents.

Variable	Shenzhen (Total)	Xixiang District	Shatou District	Nanwan District
Number of respondents *n* (%)	339 (100%)	89 (26.25%)	72 (21.24%)	178 (52.51%)
Gender *n* (%)				
Male	172 (50.74%)	44 (49.44%)	37 (51.39%)	91 (51.12%)
Female	167 (49.26%)	45 (50.56%)	35 (48.51%)	87 (48.88%)
Age *n* (%)				
20 years	30 (8.84%)	6 (6.74%)	15 (20.83%)	9 (5.06%)
21–30 years	152 (44.84%)	28 (31.46%)	33 (45.83%)	91 (51.12%)
31–40 years	95 (28.02%)	30 (33.71%)	13 (18.06%)	52 (29.21%)
41–50 years	39 (11.51%)	16 (17.98%)	7 (9.72%)	16 (8.99%)
51–60 years	21 (6.19%)	8 (8.99%)	4 (5.56%)	9 (5.06%)
61 years	2 (0.60%)	1 (1.12%)	0 (0.00%)	1 (0.56%)
Education *n* (%)				
Middle school and below	102 (30.09%)	40 (44.95%)	21 (29.17%)	49 (27.53%)
High school	123 (36.28%)	34 (38.20%)	32 (44.44%)	51 (28.65%)
Bachelor	114 (33.63%)	15 (16.85%)	19 (26.39%)	78 (43.82%)
Master	0 (0.00%)	0 (0.00%)	0 (0.00%)	0 (0.00%)
Doctor	0 (0.00%)	0 (0.00%)	0 (0.00%)	0 (0.00%)
Income per month *n* (%)				
<¥5000	191 (56.34%)	51 (57.30%)	31 (43.06%)	109 (61.24%)
¥5000–10,000	110 (32.45%)	20 (22.47%)	32 (44.44%)	58 (32.58%)
¥10,000–20,000	26 (7.67%)	16 (17.98%)	4 (5.56%)	6 (3.37%)
¥20,000–30,000	7 (2.06%)	2 (2.25%)	3 (4.17%)	2 (1.12%)
>¥30,000	5 (1.48%)	0 (0.00%)	2 (2.77%)	3 (1.69%)

**Table 3:** Correlation matrix of the relations between flood risk perception, socio-demographic factors, flood risk knowledge, flood risk attitudes, and protective coping behaviors.

		1	2	3	4	5	6	7	8	9	10	11	12	13	14	15	16
1	Age	1															
2	Edu	−0.29 **	1														
3	Inc	−0.05	0.12 *	1													
4	Loc	0.06	0.07	−0.03	1												
5	FEU	−0.11 *	0.16 **	0.10	0.07	1											
6	KFT	0.10	0.16 **	0.06	0.25 **	0.19 **	1										
7	KCF	0.06	0.24 **	0.08	0.15 **	0.36 **	0.49 **	1									
8	KFD	0.07	0.12 *	0.07	0.07	0.38 **	0.35 **	0.54 **	1								
9	KSH	0.10	0.07	0.03	0.15 **	0.37 **	0.40 **	0.51 **	0.55 **	1							
10	Wor	0.10	0.04	0.09	0.13 *	0.16 **	0.12 *	0.20 **	0.16 **	0.13 *	1						
11	Tru	−0.07	0.05	0.04	−0.07	0.32 **	0.08	0.13 *	0.26 **	0.29 **	0.13 *	1					
12	PS	0.04	0.09	−0.00	0.04	0.36 **	0.15 **	0.30 **	0.29 **	0.27 **	0.15 **	0.30 **	1				
13	WCI	0.10	0.06	0.04	−0.03	0.40 **	0.25 **	0.39 **	0.39 **	0.37 **	0.11 *	0.34 **	0.43 **	1			
14	UPM	0.11 *	0.12 *	0.10	0.05	0.27 **	0.30 **	0.42 **	0.35 **	0.43 **	0.06	0.13 *	0.35 **	0.54 **	1		
15	IW	−0.19 **	0.33 **	0.14 **	−0.01	0.25 **	0.11 *	0.23 **	0.18 **	0.09	0.16 **	0.20 **	0.27 **	0.29 **	0.13 *	1	
16	SRP	0.04	0.23 **	0.07	0.15 **	0.31 **	0.38 **	0.52 **	0.39 **	0.47 **	0.04	0.20 **	0.25 **	0.34 **	0.43 **	0.20 **	1

Note: ** Correlation is significant at the 0.01 level (2-tailed); * Correlation is significant at the 0.05 level (2-tailed).

**Table 4:** Results of structural equation model (SEM) fit.

Index	Evaluation Criterion	Final Model	Judgment
χ^2^/*df*	<3.00	1.409	Satisfied
RMSEA	<0.05	0.035	Satisfied
GFI	>0.90	0.955	Satisfied
CFI	>0.90	0.970	Satisfied
TLI	>0.90	0.959	Satisfied

**Table 5:** Results of standardized indirect effects in SEM.

Latent Variables	Socio-Demographic Factors	Flood Risk Perception	Flood Risk Knowledge	Flood Risk Attitudes	Protective Coping Behaviors
Flood risk perception	0.000	0.000	0.000	0.000	0.000
Flood risk knowledge	0.653	0.000	0.000	0.000	0.000
Flood risk attitudes	0.517	0.533	0.000	0.000	0.000
Protective coping behaviors	0.489	0.636	0.593	0.000	0.000

Note: The indirect effects between two variables is multiplied by the direct effect of the path connecting these two variables.

**Table 6:** Results of standardized total effects in SEM.

Latent Variables	Socio-Demographic Factors	Flood Risk Perception	Flood Risk Knowledge	Flood Risk Attitudes	Protective Coping Behaviors
Flood risk perception	0.768	0.000	0.000	0.000	0.000
Flood risk knowledge	0.653	0.850	0.000	0.000	0.000
Flood risk attitudes	0.517	0.673	0.627	0.000	0.000
Protective coping behaviors	0.489	0.636	0.593	0.945	0.000

Note: The total effects are the sum of the indirect and direct effects between the two variables.
